# A Measure of Total Research Impact Independent of Time and Discipline

**DOI:** 10.1371/journal.pone.0046428

**Published:** 2012-11-07

**Authors:** Alberto Pepe, Michael J. Kurtz

**Affiliations:** 1 Center for Astrophysics, Harvard University, Cambridge, Massachusetts, United States of America; 2 Center for Astrophysics, Smithsonian Astrophysical Observatory, Cambridge, Massachusetts, United States of America; Institute of Marine Research, Norway

## Abstract

Authorship and citation practices evolve with time and differ by academic discipline. As such, indicators of research productivity based on citation records are naturally subject to historical and disciplinary effects. We observe these effects on a corpus of astronomer career data constructed from a database of refereed publications. We employ a simple mechanism to measure research output using author and reference counts available in bibliographic databases to develop a citation-based indicator of research productivity. The total research impact (tori) quantifies, for an individual, the total amount of scholarly work that others have devoted to his/her work, measured in the volume of research papers. A derived measure, the research impact quotient (riq), is an age-independent measure of an individual's research ability. We demonstrate that these measures are substantially less vulnerable to temporal debasement and cross-disciplinary bias than the most popular current measures. The proposed measures of research impact, tori and riq, have been implemented in the Smithsonian/NASA Astrophysics Data System.

## Introduction

Measuring the research performance of scholars plays a critical role in the allocation of scholarly resources at all levels [1]–[7]. A principal “quantitative” means of measurement has long been through the use of citations [8], [9]. Citations are routinely used to evaluate the research productivity of individuals [10], [11], journals [12]–[17], universities [5], [18], and nations [19]–[21]. The use of citations to measure research performance involves several confounding factors which tend to become more important as the degree of aggregation decreases. For the evaluation of individuals, important challenges are:

### 

#### Discipline

Citation practices vary widely among various fields. Citation rates can vary between disciplines by an order of magnitude [18]; among sub-disciplines in the same discipline they can vary by a factor of two (as discussed later).

#### Co-Authorship

A paper can have an arbitrary number of authors, from one to several thousand. Should an author of a single authored paper receive the same credit for a citation as someone who has co-authors?

#### Age

The number of citations accrued by an individual scales with the square of his/her career length [10], [22]; thus, a person with a career length of 10 years will have half the citations of an equal person with a career length of 14.14 years. This age effect problem is exacerbated by the fact that the two aforementioned challenges are time dependent. For example, in the field of astrophysics, both the mean number of references and the mean number of authors have approximately doubled in the last 20 years. [23], [24].

Some of the lesser challenges associated with using citations to measure research productivity of individuals are:

#### Self-Citation

If an author cites papers by him/herself should they count as much as citations from papers by others?

#### Curation

In addition to having a database of articles and citations, one must clean and curate its data. For example, an analysis of an individual's productivity requires that one be able to exactly identify the articles written by that individual. Name changes (e.g., due to marriage) and homonyms (name clashes, where different people have the same name) can make this a serious problem.

#### Shot Noise

Sometimes an individual can, almost entirely by chance, become an author of one or more very highly cited papers, perhaps as a student. The citation distribution is a Zipf like power law, whereby some articles are cited thousands of times more than the median; clearly, there can be circumstances where a direct count of citations is not a fair representation of impact.

In a highly influential paper, Hirsch [10] proposed a pair of citation-based measures (*h, m*) which: solve the shot-noise problem, substantially improve the age problem, and help with the curation difficulty, discussed above. The Hirsch index, *h*, is the position in a citation ranked list where the rank equals the number of citations; absent shot noise *h* is obviously proportional to the square root of the total number of citations, which grows linearly with career length [10], [22]. The *m* quotient is *h* divided by career length, and is a constant throughout the career of an individual with constant productivity in a constant environment.

**Figure 1:**
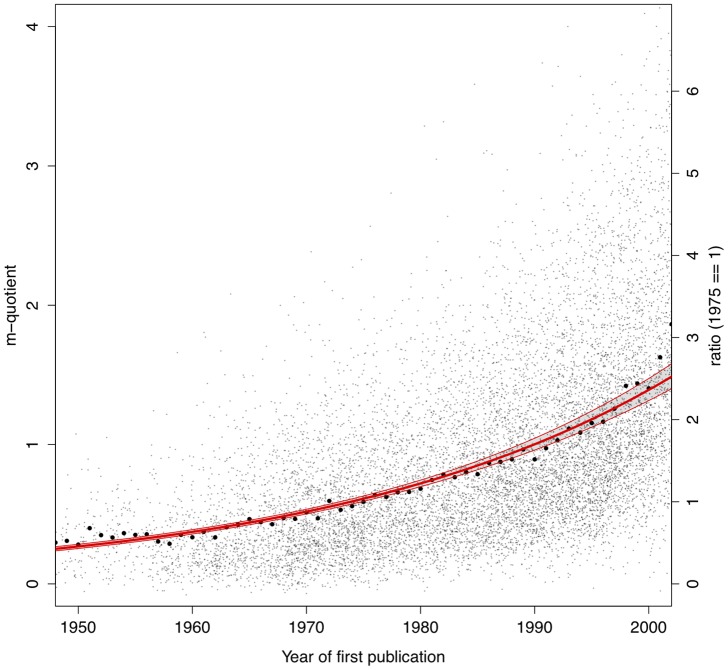
Distribution of astronomers' *m*-quotients as function of beginning of career (defined as the year of first refereed publication).

The *h*-index is by far the most widely used indicator of personal scientific productivity. As such, it has been greatly reviewed and criticized in specialized literature and innumerable alternatives have been proposed ([25], for a review). Some notable substitutes of the h-index include: the mean number of citations per paper [4], the *e*-index which complements the h-index for excess citations [7], the *g*-index, similar to *h*, but differs for it accounts for the averaged citation count an author has accrued [11], and the highly cited publications indicator [26]. Two normalizations of the *h*-index which have been proposed in the literature with promising results are by the number of article co-authors [27], and by the average number of citations per article per discipline [28]. The measures proposed in this article use both of these normalizations, combined.

**Figure 2:**
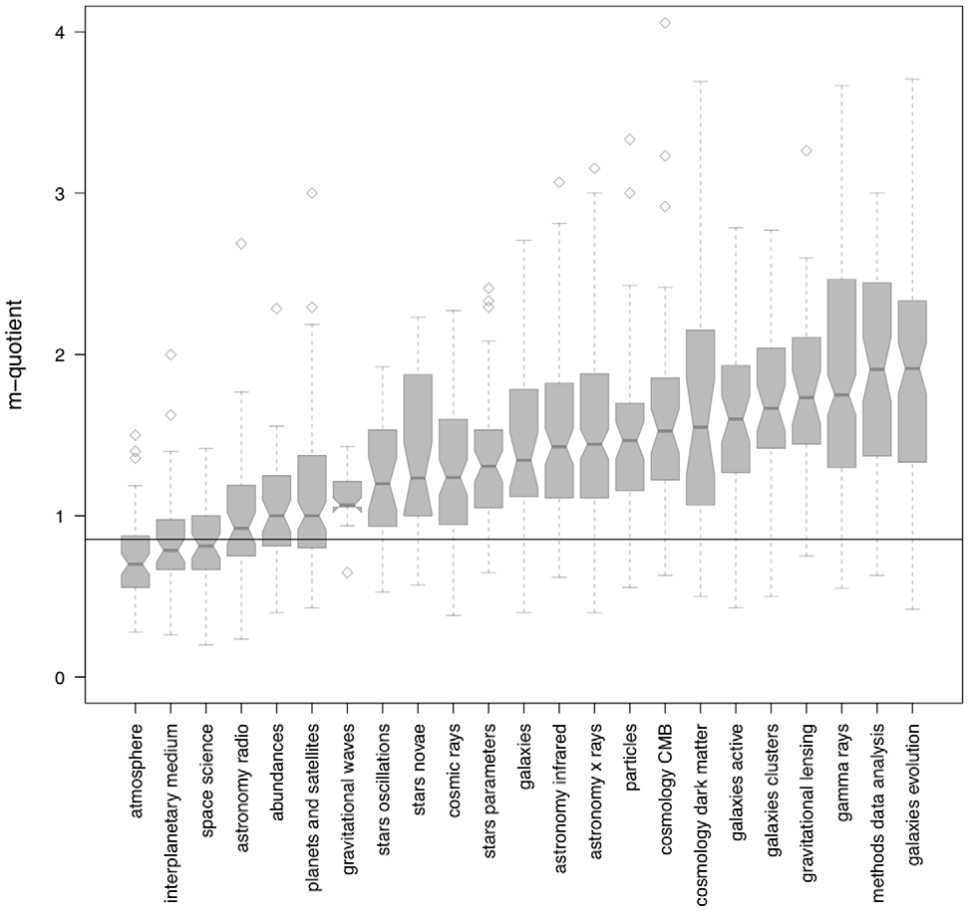
Distribution of astronomers' *m*-quotients as function of field of specialization. Each field includes between 30 and 150 astronomers who began their career in the 1990s. The box-and-whisker plot of each field depicts the median (middle notch), lower and upper quartiles (lower and upper hinges), minimum and maximum values (lower and upper whiskers), and outliers.

While the *h*-index is a valuable, simple, and effective indicator of scholarly performance, we find that it is inadequate for cross-disciplinary and historical comparisons of individuals. Comparing two scholars from different disciplines or from different time periods, or with differing co-authorship practices, based on their *h*-index would very likely yield erroneous results, simply because citation and authorship practices have changed (and constantly change) across disciplines and through time.

**Figure 3:**
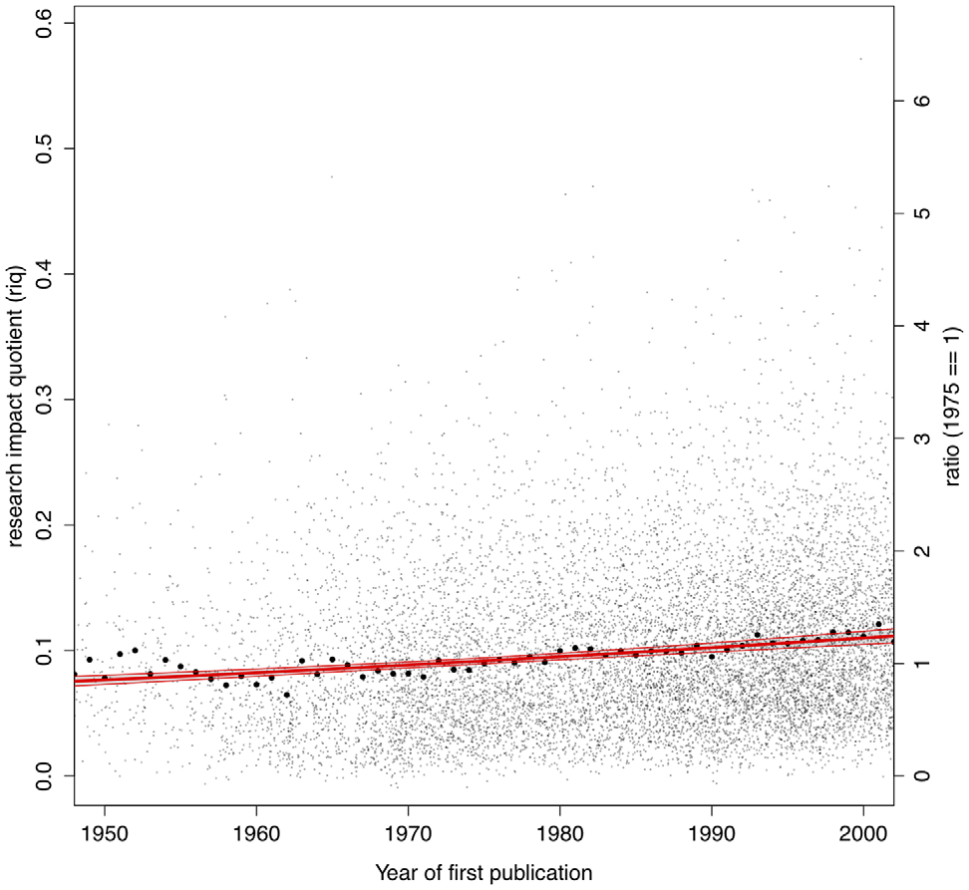
Distribution of astronomers' research impact quotients as function of beginning of career (defined as the year of first refereed publication).

**Figure 4:**
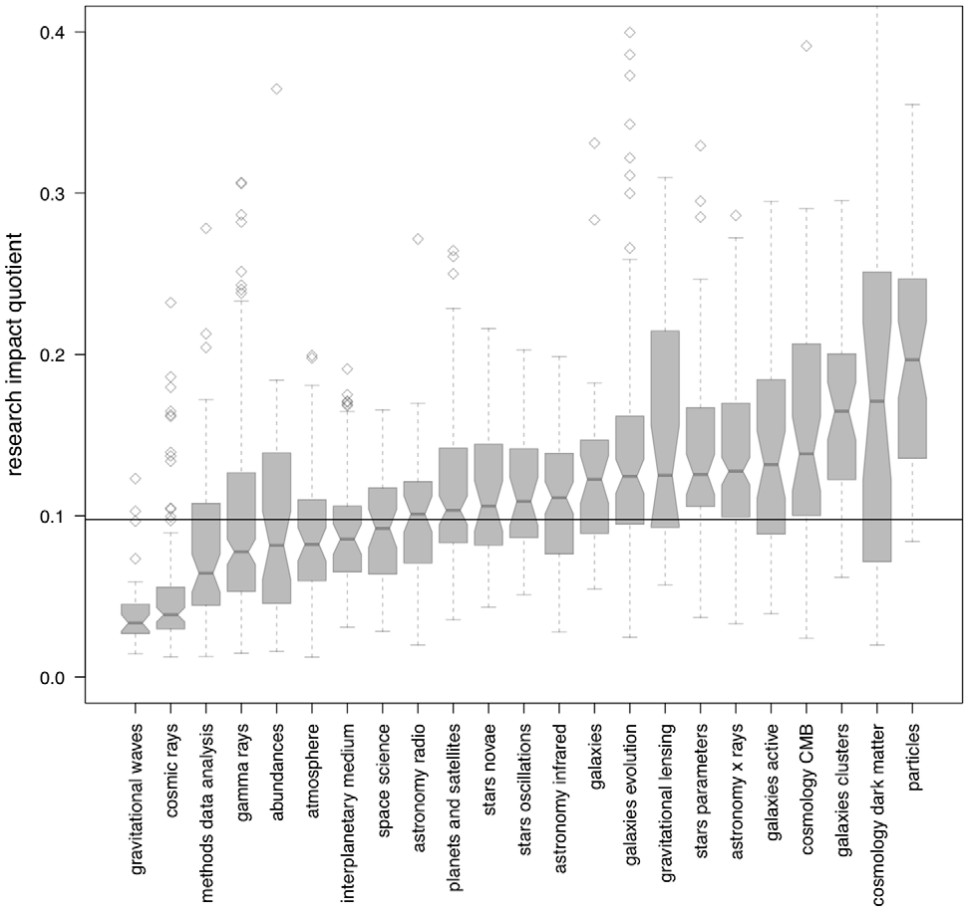
Distribution of astronomers' research impact quotient (riq) as function of field of specialization. Each field includes between 30 and 150 astronomers who began their career in the 1990s. The box-and-whisker plot of each field depicts the median (middle notch), lower and upper quartiles (lower and upper hinges), minimum and maximum values (lower and upper whiskers), and outliers.

**Figure 5:**
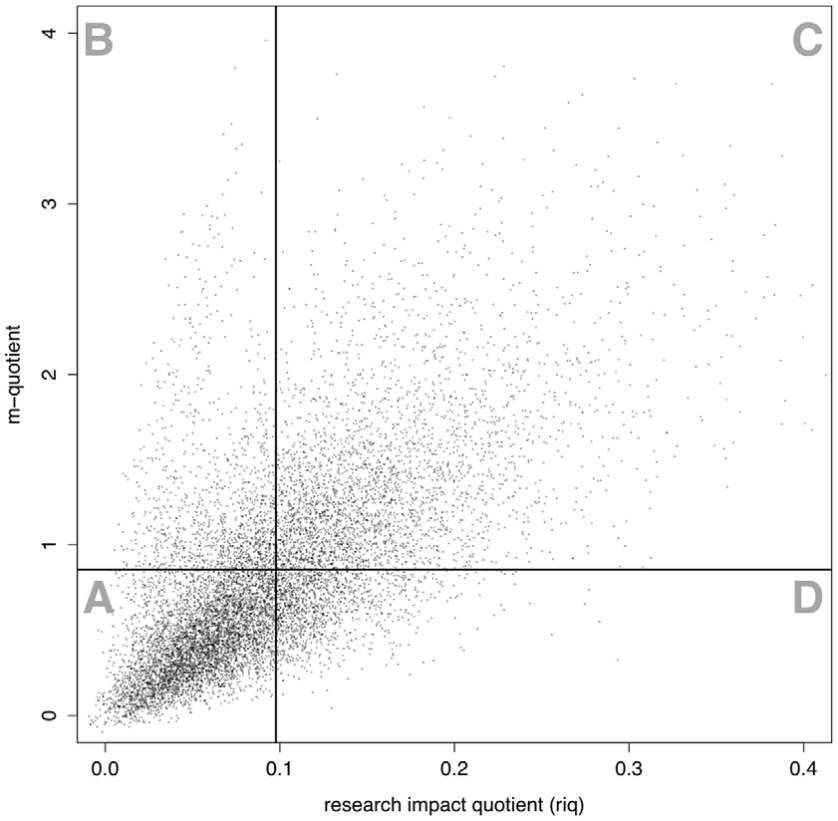
Scatterplot of *m* vs. *riq*. Horizontal and vertical lines depict the global mean *m* and *riq*, respectively.

**Table 1:** Descriptive statistics regarding astronomers with *m* above the mean and *riq* below the mean (quadrant B), astronomers with *riq* above the mean and *m* below the mean (quadrant D), and the overall population (all quadrants).

	quadrant B	quadrant D	all quadrants
size	1,221	1,300	11,036
number of publications	59.79	71.86	64.02
number of first authored publications	5.94	17.35	12.57
career start, year	1,993.1	1,977.1	1,983.5
career length, years	17.6	31.4	25.9
citations accrued, total	2,078.5	1,666.4	1,931.9
citations accrued, normalized	116.8	654.4	429.8
*h*-index	21.73	19.78	19.12
*tori*	1.81	19.66	9.55

## Methods

To investigate the historical and disciplinary effects of the *h*-index, we calculate individual researcher performance on a virtually complete astronomy database of 814,505 refereed publications extracted from the Smithsonian/NASA Astrophysics Data System (http://adsabs.harvard.edu/) [6]. We focus on the careers of 11,036 astronomers with non ambiguous names, with a publication record of over 20 refereed articles and a career span of over 10 years, who are either currently active or have a career length of at least 30 years which started on or after 1950. We define the beginning of the career as the year of publication of an astronomer's first refereed article. To begin, we compute the *m*-quotient on this cohort of astronomers and demonstrate that it is not constant over time and across sub-disciplines of astronomy. Then, we propose a novel measure of research performance, the research impact quotient (*riq*). We compute *riq* on the same bibliographic corpus showing that this derived measure eliminates most historical and disciplinary bias.

## Results

### Temporal debasement and cross-disciplinary bias of current measures

In Figure 1, we illustrate the temporal debasement of the *m*-quotient, defined as 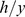, where 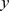 is the number of years since a scholar's first publication. Astronomers who began their career in the 1950's have systematically lower *m*s than those who started their career later on. The red line in Figure 1 is an exponential best-fit regression line with slope 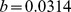 and a 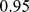 confidence interval band. Year means are plotted as filled black circles. In 50 years, the average *m*-quotient has increased from 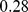 to 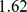, with an increase rate of 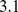
% per year, and well above the global mean of 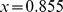. (We also run an identical regression analysis on a cohort of 697 astronomers for whom we have access to both publication record *and* Ph.D. dissertation. Using the doctoral graduation year as the starting point of their career we find similar effects of temporal debasement – best-fit regression line has slope 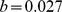).

In Figure 2 we show cross-disciplinary bias of the *m*-quotient. In the figure, the *m*-quotients of astronomers working in different fields of specialization is displayed as a box-and-whisker plot. Astronomers' fields of specialization are computed by simply selecting the single most recurrent keyword used by authors in their published articles. In order to isolate disciplinary effects, we only analyze a subset of the corpus which includes 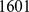 astronomers who started their career in the 1990s and who publish in popular sub-disciplines in this time window (fields with 30 authors or less are excluded from this analysis). The dashed line in Figure 2 shows the global mean *m*-quotient for all authors in the corpus (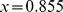). We find that for only a small portion of sub-disciplines (6 out of 23) does the global mean *m*-quotient fall within the discipline-specific upper or lower quartiles (“atmosphere” through “planets and satellites”). Astronomers who publish in all the other fields have systematically higher *m*-quotients than the global average, as evinced by higher median *m*-quotients for fields “gravitational waves” to “galaxies evolution”.

Differences so large across time and disciplines make comparison of individuals, such as in promotion and tenure decisions, quite difficult. Over time, a 3.1% yearly productivity measure inflation causes a difference of 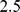 in *m*-quotient between average 40 year olds and average 65 year olds. With differences among sub-disciplines also a factor of two or more, independent of age, we suggest that citation counts and derived measures such as *h* and *m* should not be used, except for crude evaluations of scholars' impact.

### A measure of research impact independent of historical and disciplinary effects

Here we propose a novel, simple, and effective measure of research performance, designed to minimize the disciplinary and historical effects which most negatively affect citation counts and derivative measures. In addition to the volume of citations, the proposed measure employs two more bits of bibliographic information, readily available by modern scholarly databases: the number of authors and the number of references in a paper.

Both these measures have been used before, separately. Adjusting citation counts for the number of authors seems obvious [29], and has been available as an option in the ADS system since 1996 [22]. Adjusting for the number of references has become a standard technique in evaluating journals, with Web of Science using Eigenfactor [14] and SCOPUS using SNIP [15]. Similar normalizations of the *h* and other indices have been proposed in the literature, as discussed above. For example, dividing the *h*-index by the number of authors in a paper [27] and by the average number of citations per article per discipline [28] both yield promising results for cross-disciplinary impact comparison.

Thus, we normalize every external (non-self) citation received by a scholar in two ways: by the number of authors in the cited paper and by the number of references in the citing article. We speculate that *a simple double normalization, by number of authors and by number of references in the citing article, has the effect of grounding productivity index in the authorship and citation practices of a given field at a given time*.

We define the Total Research Impact, *tori*, of a scholar as:
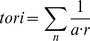
(1)where 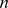 is the collection of external (non-self) citations accrued by the researcher, 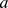 is the number of authors of the cited paper, and 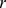 is the number of bibliographic references of the citing paper. One calculates the overall, cumulative output of a scholar by summing the impact of every external citation accrued in his/her career. As such, the total research impact of a scholar (*tori*) is simply defined as *the amount of work that others have devoted to his/her research, measured in research papers*.

The definition of *tori* influences the self-citation correction. The standard self-citation correction [30] removes a citation if any of the authors of the citing paper are the same as the authors of the cited paper. With the computation of *tori*, we only remove a citation *if the author being measured is an author of the citing paper* [31].

We can also compute the research impact averaged over a scholar's career, equivalent to the *m*-quotient. For a scholar with a career span of 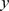 years, the Research Impact Quotient, *riq*, is defined as:
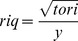
(2)


We test the performance of this measure on the same corpus discussed above, finding that the research output quotient performs very well both over time and across sub-disciplines of astronomy, as shown in Figures 3 and 4.

Temporal debasement effects are greatly attenuated when computing the *riq* on this population of scholars. As shown in Figure 3, astronomers who began their career in the 1950s do perform, on average, similar to astronomers who started publishing 50 years later (global mean is 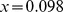). An exponential best-fit regression line (shown as solid line, with a 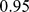 confidence band) still shows a positive gradient (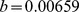), but considerably smaller than that of *m*. (A similar analysis on a cohort of 544 astronomy Ph.D. confirmed this result, finding an exponential regression line with slope 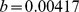). The large attenuation of temporal effects obtained with the computation of *riq* is not predominately due to either of the two normalizations: they both contribute roughly equally. The temporal slope after removing the effects of multiple co-authors is 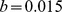 and the slope after the normalization by number of references only is 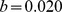.

The disciplinary bias observed for the *m*-quotient, previously discussed and depicted in Figure 2, are greatly improved when the *riq* is computed, as shown in Figure 4. While astronomers working in certain disciplines do perform below (i.e., “gravitational waves” and “cosmic rays”) or above average (i.e., “stars parameters”, “galaxies clusters”, and “particles”), the lower and upper *riq* quartile band measured for the majority of fields analyzed (18 out of 23) tends to fall within the global mean *riq* (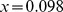, shown as a dashed line).

### Direct comparison of m-quotient and riq

To better illustrate the differences between the two measures discussed here, in Figure 5, we present a scatterplot of *m* versus *riq* for each astronomer in the corpus. The solid horizontal and vertical lines indicate the global mean for *m* and *riq*, respectively. By and large, *m* and *riq* are positively correlated, but with a substantial scatter on both sides of the main correlation trend. Moreover, an anomaly of the scatter plot is the presence of a collection of points in the upper left B quadrant: they form a branch which does not follow the main overall trend. In quadrant B, we identify 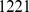 astronomers who have *m* above the global mean (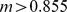), but *riq* below the global mean (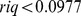). In the same way, we isolate astronomers in the lower right quadrant indicated as “D”, who have above mean *riq* and below mean *m*. Although the scatter in quadrant D is much less prominent, this group of 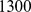 astronomers has *m* below the mean and *riq* above the mean. Astronomers in the upper left (B) and lower right (D) quadrants are interesting to explore more in detail as they are weighed very differently by the two productivity measures. Some descriptive statistics about these groups, and the overall population, are presented in Table 1.

Table 1 shows that astronomers in quadrant B and D publish differently. In quadrant D, we find astronomers who publish profusely (well above the global mean), both in general and as first authors. Astronomers in quadrant B not only publish below the mean, but also publish only a very small fraction of them as first authored works (1 in 12, as opposed to 1 in 4 for quadrant B and 1 in 5 for the overall population). Looking at the careers of astronomers in the two groups, we find that those in quadrant B tend to be younger scholars with shorter career time spans, than those in quadrant D. The citations accrued by astronomers in these two groups also follow different dynamics, with astronomers in quadrant B receiving a large volume of citations, although citation impact drops substantially below the mean if accrued citations are normalized by the number of authors in a paper. Quadrant D follows a perfectly inverse pattern: fewer overall citations, but more normalized citations. Finally, a look at the research productivity indices for the two groups reveals that quadrant B astronomers have on average higher *h* and considerably lower *tori* than the global mean (and vice versa for quadrant D). These effects, especially those relative to citation and publication, are indicative of the different archetypes of astronomers that are found in the two sections: quadrant B scholars are part of highly cited, large collaborations; quadrant D scholars are part of highly cited, yet smaller collaboration groups. A detailed examination of the careers of the individuals who are the most extreme outliers confirms this analysis.

## Discussion

The discussed measures – *tori* and *riq* – eliminate the most important systematic factors affecting the use of citations to measure the performance of individuals: the number of authors of each paper, the number of references in each paper, and the age of the individual. In addition, they remove the self citation bias. The shot noise problem is not directly addressed, however it is essentially eliminated by the number of authors correction [32]. The problem of curation was addressed in this study by careful selection of non-ambiguous names; it is being addressed more generally by initiatives such as ORCID [33].

Both *tori* and *riq* are designed to measure individuals; aggregations of individuals such as countries, universities, and departments, can be characterized by simple summary statistics, such as the number of scientists and their mean *riq*. An extension of *tori* to measure journals would be straight forward: it would consist of the simple removal of the normalization by the number of authors. The result would be similar to SNIP [15]; we suggest that SNIP and Eigenfactor [14] continue to be used for the purpose of measuring journals.

While *tori* and *riq* remove the largest systematic problems with citation counts, they are citation-derived measures and, as such, they necessarily suffer from two systematic problems of citation counts which do not lend themselves to programmatic solutions. First, it is not in general possible to tell the differing contributions of various co-authors to a paper (*tori* assumes all authors contribute equally – a technique obviously more correct in the aggregate than for any individual paper). Modifications, such as giving extra weight to the first (or last) author are necessarily ad hoc and discipline-dependent stratagems. The second fundamental problem with the use of citations for the evaluation of individuals is that citations chiefly measure usefulness [34], *not* importance; *tori* is no exception. While usefulness can be correlated with importance, these are clearly different concepts; oftentimes, importance is what is actually desired.

Measuring the research performance of scholars is a delicate and controversial procedure. That a scientists's career output cannot be condensed in a bare number is beyond discussion. Yet, providing an accurate and concise quantitative indication of a scholar's individual research output is important, and oftentimes necessary. For a faculty member at the early stages of her career, for example, a quantitative indication of her scientific productivity can be the factor determining whether she will be promoted to tenure or whether she will be awarded a research grant. In some other contexts, it may be crucial for funding bodies to know the aggregated research output of individuals working in academic institutions and scientific organizations as this can affect the course of science policy decisions.

But, most importantly, measuring research output with accuracy is important chiefly because *scholars themselves are interested in knowing their own research performance and impact*. The measures discussed here – *tori* and *riq* – while not easily computed by an individual, are easily derived from information already available in virtually all bibliographic repositories, such as Web of Science (http://wokinfo.com/), Scopus (http://www.scopus.com), SciFinder (http://www.cas.org/products/scifindr/), and ACM-DL (http://dl.acm.org/). The Astrophysics Data System (ADS) (http://adsabs.org/) has already implemented them and they are currently available to the entire community of astronomers and astrophysicists. We suggest that these measures become part other academic databases as well, to allow a fair measurement of researchers' output across time and disciplines.
